# Genome Sequences of Microviruses Identified in Gila Monster Feces

**DOI:** 10.1128/MRA.00163-21

**Published:** 2021-03-18

**Authors:** Courtney L. Collins, Dale F. DeNardo, Mellecha Blake, Jessica Norton, Kara Schmidlin, Rafaela S. Fontenele, Melissa A. Wilson, Simona Kraberger, Arvind Varsani

**Affiliations:** a The Biodesign Center for Fundamental and Applied Microbiomics, Arizona State University, Tempe, Arizona, USA; b School of Life Sciences, Arizona State University, Tempe, Arizona, USA; c Center for Evolution and Medicine, Arizona State University, Tempe, Arizona, USA; d Structural Biology Research Unit, Department of Integrative Biomedical Sciences, University of Cape Town, Observatory, Cape Town, South Africa; Portland State University

## Abstract

The complete genome sequences of 33 microviruses were determined from fecal samples collected from 14 Arizona-dwelling Gila monsters using high-throughput sequencing. These microviruses with genomes 4,383 to 6,782 nucleotides (nt) long were broadly distributed across the 14 samples.

## ANNOUNCEMENT

**FIG 1:**
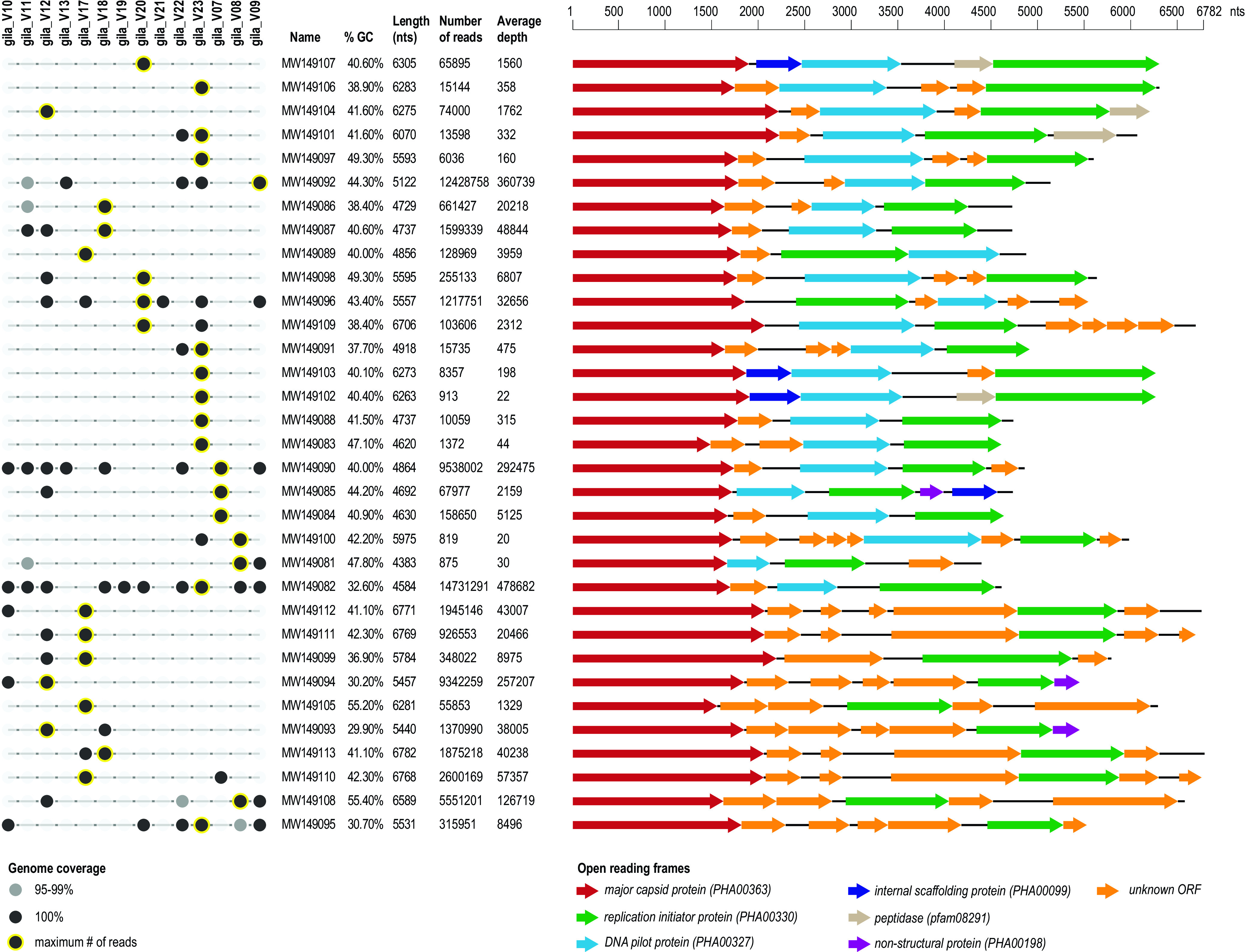
Summary of the 33 microvirus genomes identified in this study and their distribution across 14 Gila monster fecal samples. The solid dark-gray and black circles indicate 95% to 99% and 100% raw read genome coverage, respectively, per Gila monster fecal sample. The number of reads that mapped to the microvirus genome sequence and the depth of the read coverage are summarized, and the sample containing the highest number of reads for each microvirus is denoted by a black circle with a yellow outline. The genome organization is provided on the right with color-coded, open reading frames with details of putative protein families.

Gila monsters (Heloderma suspectum) are lizards found in the Sonoran Desert of North America (1). Little is known about the viruses associated with Gila monsters; to date, only adenoviruses (2, 3) and a genomovirus (4) have been reported. To further explore the viral diversity associated with Gila monsters, we analyzed the fecal samples collected directly from 14 individuals in Arizona in 2016. For each sample, 5 g of fecal material was homogenized in 20 ml of SM buffer (100 mM NaCl, 8 mM MgSO_4_, 0.01% gelatin, and 50 mM Tris-HCl) and centrifuged at 10,000 × *g* for 10 min. The supernatant was first filtered through a 0.45-μm filter followed by a 0.2-μm syringe filter. Next, 10% (wt/vol) polyethylene glycol (PEG) was added to the filtrate and incubated overnight, and the viral particles were pelleted at 10,000 × *g* for 10 min. The pellet was resuspended in 500-μl SM buffer, and 200 μl was used to extract viral DNA using the High Pure viral nucleic acid kit (Roche Diagnostics, USA). Circular DNA in the extract was amplified using rolling circle amplification (RCA) with the Illustra TempliPhi kit (GE Healthcare, USA). The RCA products were used to generate (2 × 150-bp) Illumina libraries (individually barcoded) using the Hyper prep kit (Kapa Biosystems, USA) and multiplex sequenced on a lane of an Illumina NextSeq 500 sequencer at the Arizona State University (ASU) genomics core facility. Raw reads were quality trimmed with Trimmomatic v 0.39 (5) and *de novo* assembled using metaSPAdes v 3.12.0 (6). Contigs of >1,000 nucleotides (nt) were analyzed using VirSorter (7) for bacteriophage-like sequences (including microviruses). We identified 33 unique microvirus genomes across the 14 samples. They were determined to be circular based on terminal redundancy. Given that the same genome was identified in multiple samples, we mapped the reads derived from each fecal sample to the 33 unique microvirus genomes using BBMap (8) to determine the distribution of microviruses across the samples using a threshold of 95% genome coverage for the purpose of this study (Fig. 1). All bioinformatic tools were run with default parameters.

*Microviridae* is a family of single-stranded DNA bacteriophages (9) that are found in a wide range of environments, such as seawater and animal gut samples (10–18). Microviruses have small, *T* = 1, icosahedral capsids (9) and have two classified subfamilies, namely, *Gokushovirinae* and *Bullavirinae*. The 33 microvirus genome sequences (4,383 to 6,782 nt) identified in this study have GC contents of 29.9% to 55.4% (Fig. 1). The open reading frames were identified using RASTtk (19) and annotated based on BLASTP (20) similarities to proteins encoded by microvirus sequences available in GenBank. They all encode at least a monocyte chemoattractant protein (MCP), a replication initiator protein, and their genomes have an average read fold depth ranging from 20 to 478,682 (Fig. 1). These microviruses have variable distribution across the samples (based on 95% genome coverage) ranging from 1 to 10 (Fig. 1). The MCP sequences, when analyzed with those of other microviruses available in GenBank (as of 7 December 2020), share pairwise identities in the range of 33.7% to 100% amino acid identity, as determined by SDT v 1.2 (21).

### Data availability.

The sequences of microviruses in this study have been deposited in the NCBI SRA database under project PRJNA667500 and in GenBank under the accession numbers MW149081 to MW149113.
